# Are postprandial glucose responses sufficiently person-specific to use in personalized dietary advice? Design of the RepEAT study: a fully controlled dietary intervention to determine the variation in glucose responses

**DOI:** 10.3389/fnut.2023.1281978

**Published:** 2023-12-13

**Authors:** Monique Daanje, Els Siebelink, Frank Vrieling, Maartje van den Belt, Sandra van der Haar, Johanna C. Gerdessen, Sander Kersten, Diederik Esser, Lydia A. Afman

**Affiliations:** ^1^Division of Human Nutrition, Wageningen University & Research, Wageningen, Netherlands; ^2^Food and Biobased Research, Wageningen University & Research, Wageningen, Netherlands; ^3^Department of Social Sciences, Wageningen University & Research, Wageningen, Netherlands

**Keywords:** postprandial glucose responses, person-specific meal responses, fully controlled dietary intervention, continuous glucose monitoring, study design

## Abstract

**Introduction:**

An elevated postprandial glucose response is associated with an increased risk of cardiometabolic diseases. Existing research suggests large heterogeneity in the postprandial glucose responses to identical meals and food products between individuals, but the effect of other consumed meals during the day and the order of meals during the day on the heterogeneity in postprandial glucose responses still needs to be investigated. In addition, the robustness of the glucose responses to meals or foods is still unknown.

**Objectives:**

The overall aim of the project is to assess whether the glucose response to a meal is sufficiently person-specific to use in personalized dietary advice. We aim to answer the question: “How replicable are glucose responses to meals within individuals and how consistent is the variation in glucose responses between individuals?”

**Methods:**

The question will be assessed under standardized conditions of a 9-week fully controlled dietary intervention in which all meals are the same between individuals and consumed in a fixed order at a fixed time. 63 apparently healthy men and women with a BMI of 25–40 kg/m^2^ and aged 45–75 years were enrolled in the RepEAT study (NCT05456815), of whom 53 participants completed the study. The RepEAT study comprised a fully controlled dietary intervention of nine weeks, consisting of three repetitive periods of three weeks. Within each three-week period, a variety of meals and food products were offered during breakfast, lunch, dinner and in between meal snacks. Throughout the dietary intervention, glucose was continuously monitored using Freestyle Libre Pro IQ monitors. Physical activity was monitored using the ActiGraph and ActivPAL. To measure the association between glucose responses and an individual’s phenotype, various measurements were performed before the start of the dietary intervention including an oral glucose tolerance test, a high-fat mixed meal challenge, assessment of body fat distribution including liver fat (MRI/MRS), and cardiometabolic markers.

**Discussion:**

The repetitive and fully controlled nature of the dietary study allows detailed assessment of the replicability of the glucose responses to meals and food products within individuals. Furthermore, the consistency of the variation between individuals independent of insulin resistance will be determined.

## 1 Introduction

The incidence of chronic metabolic diseases and associated healthcare costs are rising worldwide, and despite considerable attention, are expected to further increase in the coming decades (1). A common consequence of obesity and an early feature of chronic metabolic diseases is impaired glucose homeostasis as shown by elevated fasting or postprandial glucose levels (2). Elevation of postprandial glucose responses is indicative of insulin resistance and impaired glucose tolerance and is associated with an increased risk of the development of diabetes (3, 4). To correct and prevent impaired glucose homeostasis, a combination of diet- and lifestyle interventions is recommended. General dietary guidelines aim to promote health and reduce the risk of chronic metabolic diseases such as diabetes among the general adult population (5). However, although adherence to the general dietary guidelines will reduce disease risk at a population level, the current “one-size-fits-all” strategy may not provide optimized dietary advice at the individual level, by failing to take into account inter-individual differences in response to diet and meals.

Existing research shows a large heterogeneity in the plasma response to meals. Zeevi and colleagues (6) studied the variation in postprandial glucose responses in a cohort study (*n* = 800) using continuous glucose monitors. Participants followed their habitual diet and recorded consumed meals and food products in a food diary. The study showed marked variation in postprandial glucose responses between individuals to similar meals. Data of this study was used to develop an algorithm that could predict postprandial glucose responses based on physiological characteristics. The algorithm was validated in a separate cohort (*n* = 100) and dietary advice based on this algorithm could reduce postprandial glucose responses in another 26 participants. In an American cohort, the same algorithm could more accurately predict postprandial glucose responses compared to regular approaches based on nutritional content only (7). Moreover, the study of Zeevi and colleagues in 800 individuals (6) reported highly divergent responses between individuals as illustrated by one participant showing a high glucose response to a banana but not to a cookie, whereas another participant showed a high glucose response to a cookie but not a banana. Similarly, the same research group found highly divergent responses to sourdough and white bread among 20 healthy individuals (8). Collectively, these findings suggest that there might be a person-specific postprandial glucose response to meals.

More recently, Berry and colleagues (4) similarly investigated the variation in postprandial glucose responses between individuals. In this cohort study of 1,002 healthy adults, numerous baseline measurements were performed, after which glucose concentrations were continuously monitored for two weeks. Participants followed their habitual diet and consumed several standardized meals of different macronutrient compositions. The study revealed a high heterogeneity in postprandial glucose responses between individuals. Postprandial glucose responses could be predicted by a machine-learning model based on genetics, meal macronutrient composition, meal timing, sleep, exercise, and faecal microbiome composition. In addition, insulin sensitivity will affect glucose responses (9) and other physiological factors such as physical activity (4, 10) and sleep (4, 11, 12) could also influence postprandial glucose responses.

The studies of Zeevi and Berry and colleagues have revealed substantial variations in the glucose response to self-reported and standardized meals between individuals. However, several factors that may have affected the glucose responses remain to be explored. First, the time between the meal of interest and the previous meal may play a role in the glucose response. This was clearly shown in the study of Berry and colleagues (4), in which two identical meals were provided at breakfast and lunch, which resulted in a significantly higher average glucose response to the second meal at lunch compared to the first meal after an overnight fast. Standardized conditions throughout the day are essential to determine whether the glucose response to a meal is person-specific or due to consumption during a different time of the day, e.g., during breakfast or lunch. Second, previous research suggests the presence of a second-meal effect (13, 14), which is the effect of the composition of the previous meal on the next meal. Third, assessing food intake through a food diary gives a broad overview of the consumed foods, but differences in food products are not recorded. For instance, the ripeness of a banana is not recorded but may influence the postprandial glucose response. A fully controlled standardized diet with repeated consumption of the same meals and foods at a fixed time and order is essential to reliably determine person-specific postprandial glucose responses to meals, which is necessary for accurate personalized dietary advice.

The RepEAT study was designed to take all these factors into account. The overall aim of the study is to assess whether the glucose response to a meal is sufficiently person-specific to use in personalized dietary advice. We aim to answer the following question: “How replicable are glucose responses to meals within individuals and how consistent is the variation in glucose responses between individuals under standardized conditions of a 9-week fully controlled dietary intervention?”. More specifically, for example, we will answer the question: “Do individuals, compared to their average glucose response over nine weeks, replicably show large postprandial glucose responses following consumption of certain foods, whereas other individuals replicably show large postprandial glucose responses following consumption of other foods?”. In addition, we aim to examine if this variation in glucose responses is related to an individual’s phenotype.

## 2 Methods

### 2.1 Study design

The RepEAT study was a fully controlled dietary intervention trial carried out at Wageningen University & Research (WUR), Netherlands. The study was conducted between August and December 2023, and the project is currently in the phase of data cleaning. The study was approved by the Medical Ethics Committee Oost-Nederland, Netherlands (NL80179.091.21) and was registered at ClinicalTrials.gov (NCT05456815). The RepEAT study was conducted according to the Declaration of Helsinki (15), and all participants signed a written informed consent prior to the start of the study.

**Figure 1:**
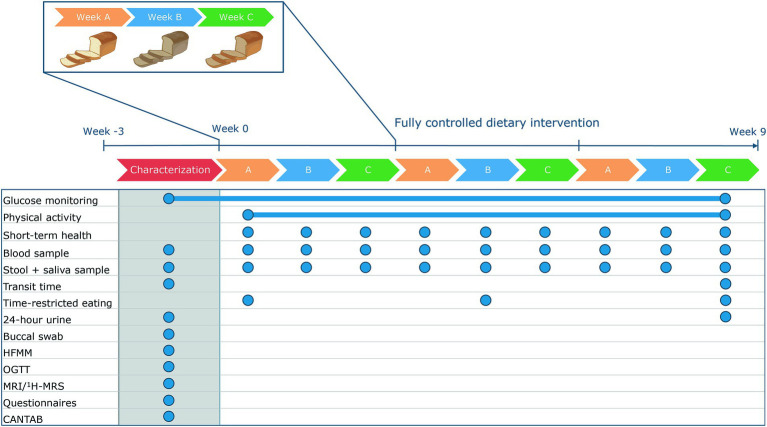
Design of the RepEAT study. In a characterization period, phenotype was identified. Afterwards, participants followed a 9-week fully controlled dietary intervention in which glucose concentrations and physical activity were continuously monitored. The dietary intervention consisted of three repetitive periods of three weeks. At the end of the 9-week dietary intervention, some of the measurements were repeated. The blue circles indicate the sample frequency. CANTAB, Cambridge neuropsychological test automated battery; HFMM, high-fat mixed meal; MRI, magnetic resonance imaging; ^1^H-MRS, proton magnetic resonance spectroscopy; OGTT, oral glucose tolerance test.

This fully controlled dietary trial consisted of a 9-week dietary intervention (Figure 1). The primary outcome of the study is the person-specific glucose responses as measured by the 2 h incremental area under the curve (iAUC). Glucose responses will be measured using continuous glucose monitoring. Glucose was continuously monitored throughout the 9-week dietary intervention to determine glucose responses to all food products and meals during the intervention. To prevent an effect of preparation of meals on the glucose response, all meals and food products were prepared and provided by the research facility. To enable the assessment of postprandial glucose responses to all meals and food products without interference of other meals, all meals and food products were consumed according to a fixed schedule with at least a two-hour interval between all meals and snacks. Physical activity was continuously monitored throughout the dietary intervention to assess physical activity patterns. To determine potential associations between an individual’s phenotype and their postprandial glucose response, participants were comprehensively characterized before the start of the dietary intervention. To assess whether consumption of a controlled diet for nine weeks reduced the overall variation in these characteristics, some of the baseline measures were repeated at the end of the dietary intervention (Figure 1).

#### 2.1.1 Study participants

Participants were recruited from a volunteer database of the Division of Human Nutrition and Health, a commercial database and via local and online advertisements and distributed flyers in Wageningen and surroundings. Apparently healthy individuals were included if they were aged between 45–75 years, had a body mass index between 25–40 kg/m^2^, and were weight stable, meaning <3 kg fluctuation in the two months prior to the screening. Exclusion criteria were among others: diagnosis of diabetes mellitus type I or type II; medication interfering with glucose metabolism and/or immune function; diseases or prior surgeries affecting the stomach, liver, or intestine; food allergies/intolerances for products used in the study design; dietary habits interfering with the study; anaemia defined as Hb concentrations <8.5 mmol/L for men and < 7.5 mmol/L for women; and veins not suitable for a venflon needle. A complete list of inclusion and exclusion criteria is provided in Supplementary Table S1. During the screening visit eligibility was assessed, where individuals filled out questionnaires to check inclusion and exclusion criteria. Hb concentrations were measured with a point-of-care finger prick test and the suitability of veins was assessed by a research nurse.

#### 2.1.2 9-week controlled diet

The 9-week diet followed the average dietary pattern of middle-aged men and women in the Netherlands according to the Dutch National Food Consumption Survey 2012–2016. The 9-week diet was designed to maintain a stable body weight, and was not designed to induce any phenotypical changes in an individual. The diet was calculated for eight energy groups ranging from 7–14 MJ / 1,600–3,400 kcal to maintain body weight stability throughout the dietary intervention. The total energy content was adjusted to energy requirements, but was similar in macronutrient composition for all energy groups, i.e., all energy groups received the same energy percentage of each macronutrient.

To design the menu cycle, first the requirements of the diet were specified, including products and meals of interest, restricted time schedules, and portion sizes. These requirements were used in a tailored version of a mixed-integer linear programming model (16) to generate a menu cycle that complied with all constraints. The nutritional composition of the diet was calculated using the Dutch Food Composition Database (NEVO-online version 2021/7.1, RIVM, Bilthoven, Netherlands). The dietary intervention consisted of three repetitive periods of three weeks. Consequently, all food products and meals were given at least three times. Within each three-week period, different versions of the same food product and meal were given at the same eating moment. For instance, participants consumed whole grain wheat bread, refined grain wheat bread, and sourdough bread on the same weekday and the same time of day.

Before the start of the dietary intervention, participants filled out a Food Frequency Questionnaire (FFQ) (17). The results of the FFQ in combination with the Schofield equations (18) were used to estimate a participant’s habitual energy intake and basal metabolic rate, respectively. Based on these calculations, participants were allocated to one of the eight energy groups to maintain body weight stability. To further support weight stability, research dieticians recorded the body weight of participants twice a week. If necessary, participants switched to a higher or lower energy group to remain weight stable.

In total, 90%–95% of the diet was provided by the research facility. To enhance compliance by giving participants some autonomy, 5%–10% of the diet was part of a “point system” in which participants chose products from a provided list. The food products mainly consisted of products high in mono- and disaccharides and/or containing alcohol. The amount of food that needed to be consumed was dependent on the energy group, and all food products reflecting the “points” had to be consumed within a set timeframe of one hour. Participants recorded all food products that were consumed using the “point system.” All food products and meals were provided in meal packages for at-home consumption, except for the food products from the point system. The participants received meal packages prepared and provided by the research facility. Twice a week, the participants visited the research facility for a joint dinner to enhance compliance, to provide room for questions and to monitor body weight. In addition, some food products were consumed in group video calls to ensure the food products were consumed at the scheduled time.

#### 2.1.3 Food products and meals

To measure the variation in postprandial glucose responses to different food products or meals, we selected a specific set of food products and meals to become incorporated into the controlled diet. These products varied each week in the three-week menu cycle and were selected based on previous literature (4, 6, 8) and societal relevance. For instance, we aimed to assess the variation in glucose responses to whole grain wheat bread, refined grain wheat bread, and sourdough bread. We also aimed to examine the variation in glucose responses to single food products, such as potato chips, chips from potato powder, and popcorn. The food products and meals that were directly compared, were consumed at the same weekday and the same time of day. For all selected products, either the carbohydrate content or the meal size remained similar; for potato chips, chips from potato powder, and popcorn, the carbohydrate content was kept comparable between the products, whereas for the different bread types the number of slices of bread was kept the same.

We also included products that were reformulated to be reduced in sugar content and/or glycaemic index. Within a product category, a reference product and two reformulations were tested within a three-week period, which was copied exactly in the second and third three-week period. Per reference/reformulated product, we once measured plasma glucose and insulin concentrations before and 2 h after consumption. To examine the relationship between consumption of the reformulated foods, glucose response, and perceived well-being, well-being was measured by a questionnaire before and 1, 2, and 4 h after consumption of the reformulated or reference products. Well-being was assessed by the adjusted Multidimensional Mood Questionnaire (MDMQ) (19), asking for fatigue, satisfaction, calmness, energy, the feeling of fitness, and tenseness on a visual analogue scale.

#### 2.1.4 Glucose monitoring

During the characterization and throughout the 9-week dietary intervention, interstitial glucose concentrations were continuously monitored using the Freestyle Libre Pro IQ (Abbott, Chicago, United States). The sensors of these continuous glucose monitors could store data for two weeks without exporting data to a reader. In addition, the glucose concentrations were blinded for participants which prevents participants to change their diet/consumption patterns based on their postprandial glucose responses. Sensors were replaced every two weeks, and although the diet remained controlled, no specific test products were planned one day after the replacements to let the sensors stabilise.

Postprandial glucose responses will be calculated as the incremental area under the curve (iAUC) from the earliest time point before the start of a meal until 2 h after the meal. In addition, the maximum glucose value and time to peak will be calculated using the most recent version of R. Next to the postprandial glucose response, the variation in glucose concentrations across the whole dietary intervention and over each three-week period will be determined. To calculate the variation over the nine-week and three-week periods, glucose concentrations over the respective period will be used to calculate outcomes of glucose variability, as reviewed by Rodbard (20): coefficient of variation (CV), standard deviation (SD), mean amplitude of glycaemic excursions (MAGE), mean absolute difference (MAD), and mean of daily differences (MODD).

#### 2.1.5 Physical activity

Physical activity was monitored using two different accelerometers. To assess physical activity patterns during the dietary intervention, physical activity was continuously monitored using the ActiGraph wGT3X/wGT3X-BT (ActiGraph, Pensacola, United States). The accelerometer was worn on the hip and the device was only removed during sleeping and bathing.

To measure light physical activity and time spent sedentary or standing, the ActivPAL3 micro (PAL Technologies, Glasgow, Scotland) was used for three non-consecutive weeks during the dietary intervention in addition to the ActiGraph. The ActivPAL was also used to examine the association between habitual sleep patterns and postprandial glucose responses. The ActivPAL was wrapped in a waterproof sleeve and attached to the right anterior thigh to ensure continuous data collection during day and night. Data were extracted after one week using PAL Software Suite version 7 (PAL Technologies, Glasgow, Scotland). During the wear-time of the ActivPAL, participants recorded sleep- and wake times in an online diary.

#### 2.1.6 Time-restricted eating test

Glucose metabolism has a diurnal pattern (21). Consumption of food products in the evening is associated with a higher glucose response compared to consumption in the morning (22). To examine the relationship between time of eating and postprandial glucose responses, participants underwent two days of time-restricted eating. On three Saturdays in the dietary intervention, the exact same meals were consumed during a different time window. On one Saturday, the standardized diet was consumed between 07:00–15:00, on a second Saturday between 14:00–23:00, and on a third Saturday, participants followed their regular schedule. The shift in the restricted time window for eating enables examination of the effect of eating time on postprandial glucose responses. Furthermore, we aimed to assess the relation between the glucose variability during the different time windows with the chronotype of an individual, i.e., whether the participants indicate themselves as a morning-type or an evening-type, using the reduced Morningness-Eveningness Questionnaire (rMEQ) (23).

### 2.2 Measurements

**Figure 2:**
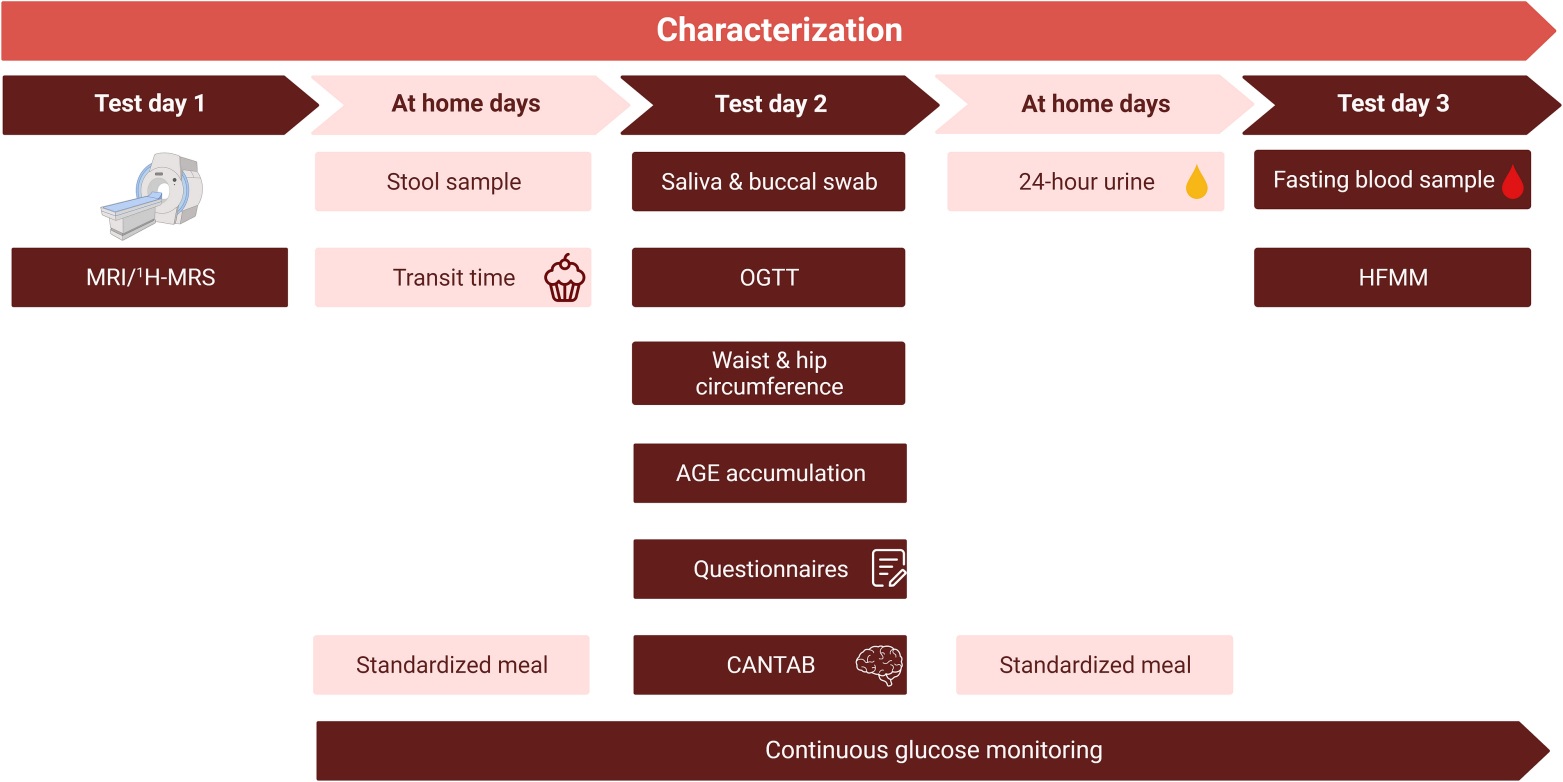
Overview of the characterization period. The characterization period consisted of three non-consecutive clinical test days and the collection of samples at home, spread over a total of three weeks. To prevent an effect of the order of the OGTT and the HFMM, the clinical test days were separated by at least one week. After a standardized meal, the evening before test days 2 and 3, participants remained fasted until arrival at the research facility. Glucose monitoring started at least 2 days before the start of test day 2. Note that the order of test days is an example, test day 2 and 3 were reversed for half of the study population. The MRI/^1^H-MRS was planned after test day 2 and 3 for some participants. CANTAB, Cambridge Neuropsychological Test Automated Battery; HFMM, high-fat mixed meal; MRI, magnetic resonance imaging; ^1^H-MRS, proton magnetic resonance spectroscopy; OGTT, oral glucose tolerance test.

Before the start of the dietary intervention, the baseline parameters of the participants were comprehensively characterized during three non-consecutive clinical test days (Figure 2). During these days several measurements were performed, including an MRI/MRS, an oral glucose tolerance test (OGTT), and a high-fat mixed meal (HFMM) challenge test. To prevent an effect of the order of the OGTT and the HFMM, the clinical test days were separated by at least one week. Glucose was continuously monitored during the OGTT and the HFMM and the six days at home in between the two tests. In addition to the characterization period, several measurements were performed during and after the intervention. An overview of all measurements performed during the RepEAT study is described in Table 1.

**Table 1:** Overview of measurements during the RepEAT study.

Outcome category	Method	Main parameters	Week		
			0	0–9	9
Anthropometrics, body composition	Weighing scale	Body weight	●	●	●
	Measuring tape	Height, waist, and hip circumference	●		
	Magnetic resonance imaging (MRI)	Abdominal subcutaneous adipose tissue, visceral adipose tissue	●		
	Proton magnetic resonance spectroscopy (^1^H-MRS)	Intrahepatic lipid content	●		
Laboratory fasting and challenge tests	Fasting blood collection	Fasting glucose and lipid metabolism	●		●
	Oral glucose tolerance test	Glucose homeostasis parameters	●		
	High-fat mixed meal	Fasting and postprandial metabolic profile	●		
Measurements in daily life	Continuous interstitial glucose concentrations	Postprandial glucose responses, glycaemic variability	●	●	
	Physical activity monitoring	ActiGraph (9 weeks), ActivPAL (3× 1 week)		●	
Microbiota composition and functionality	Faeces collection	Faecal microbiota composition	●	●	●
	Bristol stool chart	Stool frequency and consistency	●	●	●
	Blue dye method	Transit time	●		●
	Saliva collection	Oral microbiota composition	●	●	●
Immune function	SCENITH*	PBMC metabolic profile**	●		●
	Transcriptomics	PBMC transcriptome	●		●
Cognitive performance	Cambridge Neuropsychological Test Automated Battery	Attention & psychomotor speed, memory, executive function	●		
Genetics	Buccal swab/buffy coat collection	Genetic variation in genes relevant to metabolism and response to food	●		●
Amylase	Saliva collection	Amylase concentration	●	●	●
Questionnaires	General questionnaire	Demographics, medication use, lifestyle	●		
	Food frequency questionnaire	Habitual dietary intake	●		
	Perceived stress scale-10	Perceived stress	●		
	Reduced morningness-eveningness questionnaire	Chronotype	●		
	Pittsburg Sleep Quality Index	Sleep quality	●		
	Multidimensional Mood Questionnaire	Self-reported mood		●	

*SCENITH: Single Cell Energetic metabolism by profiling Translation inhibition. **PBMC: peripheral blood mononuclear cells.

#### 2.2.1 Anthropometry

The body weight of participants was measured using a calibrated digital scale to the nearest 0.1 kg. Height was measured using a telescopic measuring tape to the nearest 0.1 cm. Participants did not wear shoes or heavy clothes during both measurements. Waist and hip circumference were measured in duplicate to the nearest 0.1 cm.

#### 2.2.2 OGTT and HFMM

To characterize the metabolic response to a meal challenge and to determine insulin resistance, a 7-point oral glucose tolerance test (OGTT) and a high-fat mixed meal (HFMM) test were performed once per individual in the characterization period. The evening before both the OGTT and the HFMM, participants consumed a standardized dinner (24% of energy intake (en%) fat, 54 en% carbohydrates, 22 en% protein; in total 2,380 kJ/565 kcal). After the standardized dinner participants remained fasted until arriving at the research facility. In addition, participants refrained from heavy exercise the evening before and the morning of these tests. Thirty minutes before the start of the test, a venous catheter was placed.

For the OGTT, at *t* = 0 min, participants drank 200 mL of a 75 g glucose solution (Glucomedics, Breda, The Netherlands) within five minutes. Blood was collected in the fasted state before (*t* = 0), and 15, 30, 45, 60, 90, and 120 min after consumption of the glucose solution, to measure plasma glucose and insulin concentrations. The HFMM was provided in the form of a shake and consisted of 320 g water, 20 g protifar, 83.5 g dextrose, 60 g palm olein, and 20 drops of vanilla oil. In total, the shake contained 3,833 kJ and 76.3 g carbohydrates, 17.6 g protein, and 60 g fat (24). To make sure there was no difference in metabolic response due to a difference in consumption speed, participants were instructed to consume the drink in 10 min. Blood samples were collected before consumption (*t* = 0) and 30, 60, 90, 120, 180, and 240 min after the consumption of the HFMM for the measurement of plasma glucose and insulin, free fatty acids, short-chain fatty acids, triglycerides, cholesterol, gut hormones, metabolomics, and proteomics including cytokines. At each time point, the first 2 mL of blood was discarded before drawing the blood samples because it could still contain the physiological salt of the venflon line.

#### 2.2.3 Fasting blood outcomes

Fasting blood samples were collected at baseline, before the start of the OGTT or HFMM, and at the end of the 9-week dietary intervention. Fasting blood was collected to measure glucose homeostasis, oxidative stress markers, plasma concentrations of advanced glycation end-products (AGEs), and immune cell metabolism. We aimed to determine fasting glucose homeostasis by measuring plasma glucose, insulin, and glycated haemoglobin (HbA1c) concentrations after an overnight fast. In addition, fasting blood samples were collected to measure concentrations of oxidative stress markers, and to determine the plasma concentrations of α-dicarbonyls and advanced glycation end-products (AGEs) using ultraperformance liquid chromatography–tandem mass spectrometry. Furthermore, fasting blood samples were used to isolate peripheral blood mononuclear cells (PBMCs) and perform immune cell metabolic profiling using an adapted version of SCENITH (Single Cell Energetic metabolism by profiling Translation inhibition) (25). Additionally, peripheral blood mononuclear cells were stored in Trizol for transcriptomic analysis.

#### 2.2.4 24-h urine

Before and at the end of the 9-week dietary intervention, 24-h urine was collected to measure oxidative stress markers. Participants collected 24-h urine in 3 L containers at home. The measurement started after the first voiding in the morning and ended 24 h later. At the day of urine collection, participants ingested a tablet containing 100 mg of para-aminobenzoic acid (PABA) during during breakfast, lunch, and dinner, to check whether all urine was collected. Participants were asked to store the urine samples in a cool place until turning them in the next day. Urine samples per participant were mixed, the volume was measured, and aliquots were stored at −80°C until further analysis.

#### 2.2.5 Skin autofluorescence

Next to the plasma concentrations of AGEs, the accumulation of AGEs in the skin was measured by skin autofluorescence at 440 nm using an AGE reader (Diagnoptics, Groningen, Netherlands) (26). This is a non-invasive procedure in which the participants rested their lower arm on the AGE reader for 12 s during which the skin autofluorescence was measured. Participants were instructed to not apply any body cream or sunscreen on the day of the measurement. The measurement was performed on a skin site without skin abnormalities such as scars, tattoos, or birthmarks.

#### 2.2.6 Magnetic resonance imaging/spectroscopy

Magnetic resonance imaging (MRI) was used to measure abdominal fat distribution and to guide spectroscopic measurements for quantification of intrahepatic lipid (IHL) content. The procedures were performed on a Philips Ingenia Elition 3 T X MRI scanner (Royal Philips, Amsterdam, Netherlands). Participants were asked to refrain from eating and drinking two hours before the start of the scan to ensure that digestion did not interfere with the measurements. The scans were performed in the MRI/MRS-scanner using a body coil with the participant in a supine and head-first position. Breath-hold techniques on exhalation were used to avoid breathing-induced artefacts.

To measure abdominal fat distribution, axial T1-weighted spin echo images were acquired and slices were centred at the interspace L3-L4. Visceral (VAT) and subcutaneous adipose tissue (SAT) and their ratio were quantified using the software Slice-O-Matic version 6 Rev-7. To quantify the intrahepatic lipid (IHL) content, proton magnetic resonance spectroscopy (^1^H-MRS) was used. Fine shimming was performed to optimize the magnetic field homogeneity within the region of interest and a 30x30x20 mm voxel was placed in the upper right liver lobe. Vascular structures and the proximity of subcutaneous fat were avoided during the voxel placement. The water signal in the ^1^H-MRS spectra was suppressed using frequency-selective pre-pulses and the spectra were fitted to quantify the lipid peak. Liver measurements were triggered 500 ms following inhalation. The ^1^H-MRS spectra were processed and the IHL content was quantified by the AMARES algorithm in jMRUI software v5.2.

#### 2.2.7 Saliva and buccal swabs

To determine the oral microbiota composition and to measure salivary amylase activity, saliva was collected at baseline and every week during the dietary intervention. Saliva was collected by the passive drool collection method (Salimetrics, Carlsbad, United States). After rinsing their mouth, participants sat in a comfortable position with their heads slightly tilted forward. Saliva was collected by slowly and passively guiding the saliva to the collection tube. Samples were stored at −20°C until further use. Salivary amylase activity was measured using a colorimetric assay (Salimetrics, Carlsbad, United States). Next to the saliva measurements, a buccal swab and/or buffy coat were collected to isolate DNA to measure the copy number variation of genes encoding for salivary and pancreatic amylase: *AMY1, AMY2A,* and *AMY2B* and for future analyses of genetic variation related to glucose and insulin responses. DNA from the buccal swab was isolated using NAOH extraction, and DNA from the buffy coat was isolated using the Blood Mini Kit (Qiagen, Hilden, Germany). Digital droplet polymerase chain reaction (ddPCR) was performed and the copy number of each gene was determined using a reference gene *RPP30* with a known copy number of 2.

#### 2.2.8 Intestinal tract measures

Before the start of the dietary intervention and every week during the dietary intervention, faecal samples were collected to measure the faecal microbiota composition. On the day of faecal sample collection, participants were asked to indicate stool frequency of the past week and to rate stool consistency using the Bristol Stool Chart (27). In addition, transit time was measured using the “blue dye method” (28). During the characterization period and at the end of the 9-week dietary intervention, participants consumed two cupcakes containing in total 1.5 g of blue colouring paste (for consumption purposes) within 10 min. The date and time of the first appearance of the blue dye in the faeces were recorded by participants and were used as a measure of transit time.

#### 2.2.9 Questionnaires

During the characterization period, participants filled out three questionnaires to assess perceived stress, chronotype, and sleep quality. The Perceived Stress Scale (PSS-10) contained 10 questions about the perceived stress level of participants in the past month (29, 30). The rMEQ (23) was used to assess the chronotype of an individual. In other words, people indicated themselves as a “morning personality type” or an “evening personality type.” Sleep quality over the past month was measured by the Pittsburg Sleep Quality Index (PSQI) (31).

#### 2.2.10 Cognitive performance

To examine cognitive performance at baseline, participants performed the Cambridge Neuropsychological Test Automated Battery (CANTAB) consisting of a series of small tests. Before the start of the test battery, participants consumed a standardized meal without caffeine. The tests were performed on a tablet with a touch screen, in a quiet environment to prevent distraction. Participants received a short introduction and underwent a practice round before the start of each test to familiarize themselves with the test. The series of tests took 45–60 min. In total, six tests of the CANTAB gave insight into the performance of three distinct cognitive domains: attention and psychomotor speed, executive function, and memory.

#### 2.2.11 Compliance

To increase compliance, participants had to visit the facility twice a week to have a joint dinner under supervision of the researchers and to have the opportunity to ask questions to a research dietician. Two days a week, subsamples of test products were consumed via video calls to monitor compliance. Compliance was optimized by adjusting the restricted eating schedule to an individual’s needs, taking into account regular waking and sleeping times. Furthermore, the free point system enabled participants to choose some products of their preference. Compliance was monitored using the LifeData application (LifeData, Marion, United States) by filling out a short daily questionnaire that asked for any deviations in product consumption, consumption times, and exercise patterns.

### 2.3 Data management

Data were collected and stored in an electronic case report form (eCRF) using the data capturing platform Castor EDC (Castor EDC, Amsterdam, Netherlands), which is in accordance with Good Clinical Practice regulations. All protocol deviations and adverse events were recorded via the eCRF.

### 2.4 Sample size calculation

**Figure 3:**
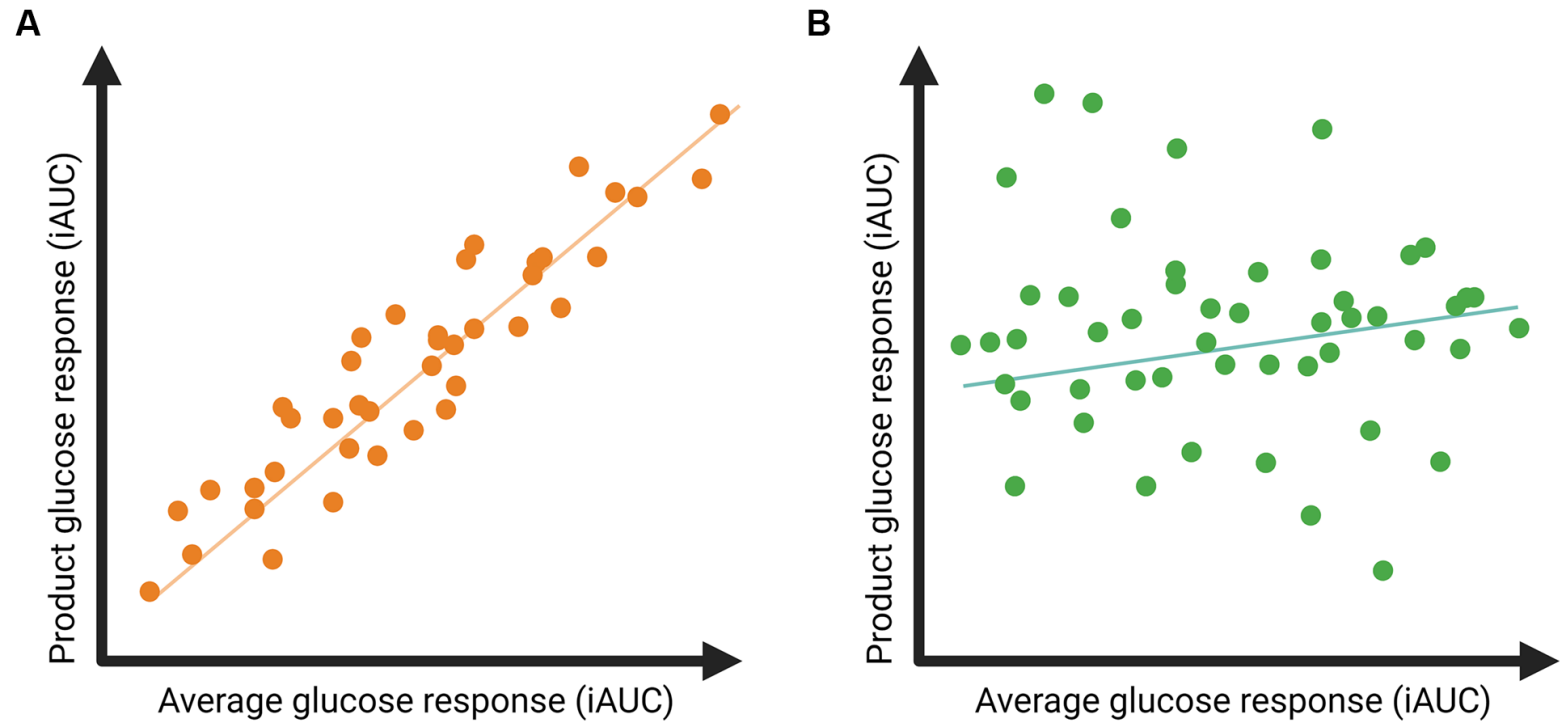
Example of expected results regarding postprandial glucose responses to a specific food product related to the individual’s average glucose response. **(A)** A high correlation indicates that postprandial glucose responses to the product are in line with an individual’s average glucose response. **(B)** A low correlation indicates that the postprandial glucose responses to the product deviate from an individual’s average glucose response, suggesting the presence of meal-specific glucose responses.

We aim to assess whether the glucose response to a meal is sufficiently person-specific to use in personalized dietary advice and will determine person-specific responses to meals and food products in the dietary intervention. The person-specific responses will be identified by correlating the glucose response to a meal or food product to the individual’s average postprandial glucose response over the 9-week controlled diet. An example of potential results is depicted in Figure 3. A high correlation would indicate that individuals are likely to respond as expected based on the average glucose response (Figure 3A), so no person-specific response. Low correlations would indicate a person-specific response; for example, individuals with a high average glucose response may have a low glucose response to the meal or food product, and vice versa (Figure 3B).

To estimate the required sample size to be able to detect meal-specific responses, we used preliminary data from 27 participants of the GLYSIMI study (NCT05120661). The GLYSIMI study investigated the role of the human small intestine microbiota in regulating postprandial glucose responses to food products. The study included men and women with a BMI of 25–40 kg/m^2^, aged 40–75 years. During the screening phase of the GLYSIMI study, participants wore a continuous glucose monitor, and glucose responses after seven standardized products at breakfast or dinner were compared. All glucose responses were measured as the iAUC (mg/dl*hour, 120 min postprandially). The average glucose response was calculated as the average glucose response to all products per individual. Correlations between postprandial glucose responses to specific foods and the average postprandial glucose response of an individual ranged between 0.49 and 0.85.

In the RepEAT study, we expect lower correlations between glucose responses to specific food products and the average glucose response, because we measured nine weeks of a fully controlled diet and not only a few standardized meals. Therefore, we expected correlations ranging between 0.3 and 0.4. Using GPower (version 3.1.9.7) and a “Correlation: Point biserial model,” with a two-sided test, an α of 0.05, a power of 0.80, and an effect size of 0.3 and 0.4, this would lead to a required samples size of, respectively, 44 or 82 participants. We chose the average of the two and therefore 57 participants were needed in this study. Considering a 10% drop-out rate, the required sample size was *n*=57 + 6=**63** participants.

## 3 Statistical analysis

Within the RepEAT study, we aim to examine how replicable glucose responses are within individuals and how consistent the variation in glucose responses is between individuals under standardized conditions of a 9-week fully controlled dietary intervention. To determine the replicability of glucose responses to identical meals and food products within individuals, we will compare the three repetitions per meal or food product. To assess the variability in glucose responses to identical meals between individuals, we will compare the three repetitions per product between individuals. Glucose responses will be corrected for metabolic state of insulin sensitivity by using the average glucose response to all meals in the diet and/or the glucose response to the OGTT, and by correcting for physical activity.

IBM SPSS Statistics and the latest R software will be used to carry out the statistical analyses. Numerical values in descriptive statistics will be reported as mean ± SD, categorical values will be reported as numbers and percentages. To determine the normality of the data, a visual inspection of QQ plots and results of the Kolmogorov–Smirnov test will be used. For linear mixed model analyses, model residuals will be checked for normality. Non-normally distributed data will be transformed before the analyses. *p*-values <0.05 will be considered statistically significant.

Glucose responses to single meals will be measured as the incremental area under the curve (iAUC; mg/dl*hour) and will be calculated from the latest timepoint before consumption until 120 min after consumption. In addition, the glucose peak and time to peak will be measured within each postprandial glucose response curve, within 120 min after consumption of a test product. For each meal and food product there will be three repeated measures during the dietary intervention. Glucose responses to single meals will be associated with the average iAUC of all glucose responses of an individual. There will be adjusted for multiple testing using false-discovery rate adjustment according to the Benjamini-Hochberg procedure (32).

## 4 Results

**Figure 4:**
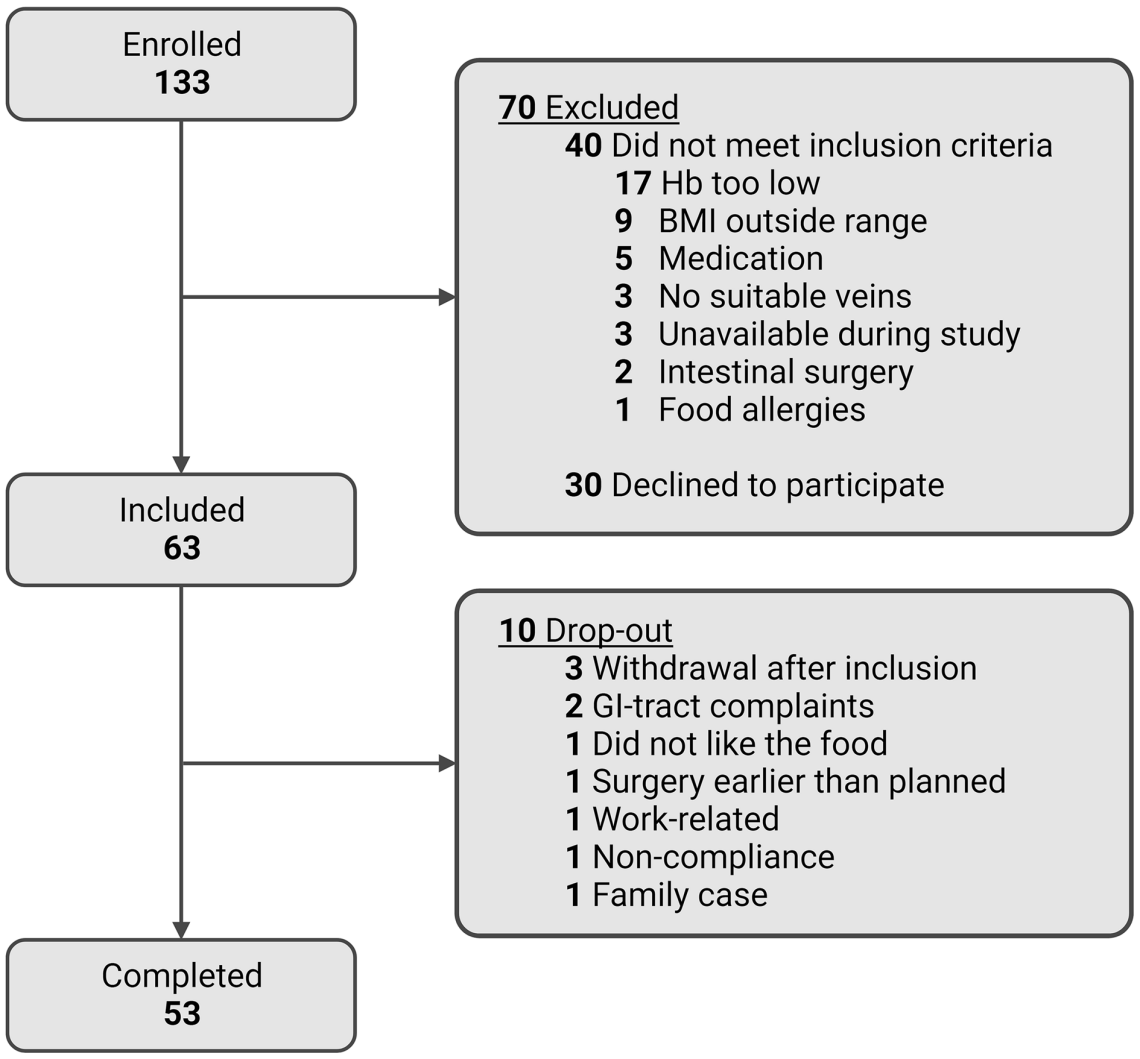
Flowchart of enrolment and completion of study participants.

Figure 4 shows a flowchart of the participant enrolment and completion of the RepEAT study. In total, 506 individuals showed interest in the study, of whom 223 were still interested and scheduled for an information session after receiving the written participant information. After having received the written and verbal information, 133 individuals signed the informed consent form and underwent the screening procedure. Of these 133 individuals, 63 individuals were eligible according to all inclusion and exclusion criteria, and were included in the study. During the study, 10 participants dropped out (15.9% drop-out rate) at different stages of the study and for different reasons (depicted in Figure 4). 53 participants completed the study, which is well within the beforehand estimated sample size range of 44–82 participants.

The baseline characteristics of all participants that started the dietary intervention (*n* = 60) are depicted in Table 2. Participants were on average 63.9 (± 8.3) years old with a BMI of 30.3 (± 4.0) kg/m^2^, and 45% of the study population were women. In total, each participant who completed the study consumed 378 meals and we collected ~6,000 interstitial glucose concentrations per individual over the 9-week dietary intervention.

**Table 2:** Baseline characteristics of participants who started the dietary intervention (*n* = 60).

Age (years)	63.9 ± 8.3
Women (*n*, %)	27 (45%)
Weight (kg)	90.6 ± 14.3
BMI (kg/m^2^)	30.3 ± 4.0
Waist circumference (cm)	106.0 ± 12.2
Waist-to-hip ratio	1.0 ± 0.1
SBP (mmHg)	128.9 ± 18.9
DBP (mmHg)	74.2 ± 10.8
Use of antihypertensives (*n*, %)	11 (18%)

Categorical data are depicted as *n* (%), numerical data are depicted as mean ± SD. BMI, body mass index; DBP, diastolic blood pressure; SBP, systolic blood pressure.

## 5 Discussion

This manuscript describes the protocol of the RepEAT study, which was designed to answer the question: “how replicable are glucose responses within individuals and how consistent is the variation in glucose responses between individuals under standardized conditions of a 9-week fully controlled dietary intervention?”. Furthermore, we aim to examine how this person-specific variation in glucose responses to meals is related to the diet, the time of consumption, and to an individual’s phenotype. To measure glucose responses in a standardized setting, the RepEAT study was a fully controlled dietary intervention study of nine weeks, including three repetitions of a 3-week menu cycle. Before the start of the dietary intervention, participants were comprehensively characterized to study associations between postprandial glucose responses and phenotype.

Up to now, most evidence regarding postprandial glucose responses and variation between and within individuals originates from cohort studies, with only a few standardized meals next to the habitual diet of participants (4, 6, 8). By making use of a fully controlled dietary intervention with continuous glucose and physical activity monitoring, the RepEAT study will provide further insight in the variation in glucose responses independent of external factors such as physical activity, previous meals, and time of day. Moreover, the degree of insulin resistance is an important factor affecting postprandial glucose responses. In the RepEAT study, insulin resistance is determined by an oral glucose tolerance test and will be used to correct for an individual’s insulin resistance status. In addition, all meals were consumed according to a schedule with at least two hours in between meals and snacks to enable us to determine the full glucose response, since on average the glucose concentration returns to baseline within two hours. Furthermore, in order to examine the variation in glucose responses to identical meals and food products within an individual, the 9-week diet consisted of three identical periods of three weeks, resulting in three repetitive measures of postprandial glucose responses to all meals and food products. Given the standardized dietary background, these three repetitive measures per meal and food products will be analysed to measure the robustness of postprandial glucose responses within an individual. Within each three-week period, different versions of the same food product and meal were consumed at the same weekday and time of day, which enables us to study glucose responses to variations of the same food product or meal.

In conclusion, with the RepEAT study we will provide an answer to the question whether person-specific glucose responses to meals are unique and robust enough to be able to be used in personalized advice.

## Ethics statement

The studies involving humans were approved by Medical Ethics Committee Oost-Nederland, Netherlands. The studies were conducted in accordance with the local legislation and institutional requirements. The participants provided their written informed consent to participate in this study.

## Author contributions

MD: Conceptualization, Data curation, Investigation, Methodology, Writing – original draft. ES: Conceptualization, Investigation, Methodology, Writing – review & editing. FV: Investigation, Methodology, Writing – review & editing. MB: Data curation, Investigation, Writing – review & editing. SH: Data curation, Investigation, Writing – review & editing. JG: Methodology, Writing – review & editing. SK: Conceptualization, Funding acquisition, Methodology, Writing – review & editing. DE: Conceptualization, Funding acquisition, Methodology, Supervision, Writing – review & editing. LA: Conceptualization, Funding acquisition, Methodology, Supervision, Writing – review & editing.
