# Supplementary material for: Are postprandial glucose responses sufficiently person-specific to use in personalized dietary advice? Design of the RepEAT study: a fully controlled dietary intervention to determine the variation in glucose responses
Source: Front Nutr. 2023 Dec 13;10:1281978. doi: 10.3389/fnut.2023.1281978 (PMC10751339; doi:10.3389/fnut.2023.1281978)
Supplement: Supplementary file 1 [file Table_1.DOCX]

Table S1. Inclusion and exclusion criteria for the RepEAT study.

| **Inclusion criteria** | - Apparently healthy men and women - BMI of 25 – 40 kg/m^2^ - Age 45-75 years - Weight stable (± <3 kg) for at least two months prior to inclusion |
| --- | --- |
| **Exclusion criteria** | - Diagnosed with type 1 or type 2 diabetes - Diseases or prior surgeries affecting the stomach, liver, or intestines - Food allergies/intolerances for products used in the study design - Receiving medication or supplements interfering with glucose metabolism (as judged by our research physician) - Regular use of medication interfering with immune function (e.g. corticosteroids, immune blockers, as judged by our research physician) - Donated blood within 2 months prior to the screening - Anaemia defined as Hb concentrations <8.5 mmol/L for men and <7.5 mmol/L for women - Veins not suitable for venflon needle - Allergy/intolerance to medical skin adhesives - Dietary habits interfering with the study design (e.g. vegetarian, vegan, ketogenic diet) - Intention to change the intensity of exercise during the study period - Current smokers - Alcohol intake ≥14 alcoholic beverages per week (women) or ≥21 alcoholic beverages per week (men) - Being pregnant or lactating - Use of soft and/or hard drugs - Unable/unwilling to download a research application on the mobile phone - Participation in another study that involves an intervention within two months prior to the screening - Working at the division of Human Nutrition and Health of Wageningen University and Research or the Food, Health and Consumer research group of Wageningen University and Biobased Research |
